# The experience of traumatic events disrupts the measurement invariance of a posttraumatic stress scale

**DOI:** 10.3389/fpsyg.2014.01304

**Published:** 2014-11-18

**Authors:** Miriam J. J. Lommen, Rens van de Schoot, Iris M. Engelhard

**Affiliations:** ^1^Experimental Psychology, University of OxfordOxford, UK; ^2^Method and Statistics, Utrecht UniversityUtrecht, Netherlands; ^3^Optentia Research Program, Faculty of Humanities, North-West UniversityVanderbijlpark, South Africa; ^4^Clinical and Health Psychology, Utrecht UniversityUtrecht, Netherlands

**Keywords:** measurement invariance, posttraumatic stress disorder, trauma, threshold instability, multiple assessments

## Abstract

Studies that include multiple assessments of a particular instrument within the same population are based on the presumption that this instrument measures the same construct over time. But what if the meaning of the construct changes over time due to one's experiences? For example, the experience of a traumatic event can influence one's view of the world, others, and self, and may disrupt the stability of a questionnaire measuring posttraumatic stress symptoms (i.e., it may affect the interpretation of items). Nevertheless, assessments before *and* after such a traumatic event are crucial to study longitudinal development of posttraumatic stress symptoms. In this study, we examined measurement invariance of posttraumatic stress symptoms in a sample of Dutch soldiers before and after they went on deployment to Afghanistan (*N* = 249). Results showed that the underlying measurement model before deployment was different from the measurement model after deployment due to invariant item thresholds. These results were replicated in a sample of soldiers deployed to Iraq (*N* = 305). Since the lack of measurement invariance was due to instability of the majority of the items, it seems reasonable to conclude that the underlying construct of PSS is unstable over time if war-zone related traumatic events occur in between measurements. From a statistical point of view, the scores over time cannot be compared when there is a lack of measurement invariance. The main message of this paper is that researchers working with posttraumatic stress questionnaires in longitudinal studies should not take measurement invariance for granted, but should use pre- and post-symptom scores as different constructs for each time point in the analysis.

## Introduction

Questionnaires are often used at different time points to assess mean or individual change over time. For example, a questionnaire to assess posttraumatic stress symptoms can be rated at different time points after a traumatic event to study the course of problematic responses. Although statisticians have stressed the importance of testing measurement invariance when comparing latent mean scores over time (e.g., Byrne et al., 1989; Steenkamp and Baumgartner, 1998; Vandenberg and Lance, 2000), the assumption that factor loadings and intercepts (or thresholds when dealing with dichotomous or categorical scores instead of continuous scores) of the underlying items are equal over time often seems to be taken for granted. By comparing latent mean scores over time, we aim to capture *true* latent score changes (i.e., alpha change; Brown, 2006). However, in case of measurement non-invariance, increases or decreases in latent mean scores may also reflect changes in the construct itself (gamma change) or changes in the measurement proportions of the indicators (beta change). Therefore, it is important that factor loadings and intercepts are “measurement invariant” to claim *true* latent score change over time and to avoid bias in the parameter estimates (Guenole, 2014). But what should one do in case of measurement non-invariance? Is it then still possible to draw meaningful conclusions or should mean scores over time not be compared? In this article we discuss a measure that, from a theoretical perspective, is expected to lack measurement invariance. In such cases the solutions of establishing partial invariance (Byrne et al., 1989) or approximate invariance (van de Schoot et al., 2013; Muthén, 2014) are not a valid solution. We will test for measurement invariance in two samples, and investigate causes of measurement non-invariance and interpretations of the results in this situation.

### The case of theoretical measurement non-invariance

The experience of a traumatic event can lead to psychological distress, which may manifest as posttraumatic stress disorder (PTSD). PTSD is characterized by re-experiencing symptoms (e.g., intrusions or nightmares related to the event), avoidance of reminders of the event, negative cognitions and mood, and hyperarousal symptoms (e.g., sleep and concentration problems; APA, 2013). One way to check the presence of PTSD symptoms is by using self-report questionnaires. Although it is often not possible to include a pre-trauma assessment of symptomatology, several prospective longitudinal studies, typically in military or firefighter samples, have done this and showed that PTSD symptoms after a traumatic event may partially be explained by symptoms endorsed at baseline (e.g., Engelhard et al., 2007b; Rona et al., 2009; Vasterling et al., 2010; Rademaker et al., 2011; van Zuiden et al., 2011; Berntsen et al., 2012; Bonanno et al., 2012; Franz et al., 2013; Lommen et al., 2013, 2014). High scores at baseline could represent symptoms that are not exclusively related to PTSD (e.g., sleep or concentration problems, negative mood; Engelhard et al., 2009b), or they may reflect already existing PTSD symptoms resulting from earlier traumatic experiences. So when prospectively studying, for instance, predictors for the development of PTSD symptoms, it seems useful to take symptoms that were already present before trauma into account.

However, it may be hypothesized that the experience of a traumatic event^1^ (APA, 2013) can actually change the way items of the questionnaire are interpreted. That is, after experiencing a traumatic event, the probability of answering “yes” to a specific questions may increase or decrease (gamma change), and the relative importance of questions may change (beta change).

Consider, for example, soldiers who complete a questionnaire for PTSD symptoms before and after deployment. Before deployment, soldiers may be instructed to rate the items in reference to a recent event that made them feel especially upset or distressed, in reference to a distressing event that bothered them the most in the last month, or without reference to a specific event. After deployment, the soldier may be instructed to fill out the questionnaire with respect to most distressing event during the recent deployment, or to rate the symptoms without reference to a specific event. Before deployment, the presence of symptoms could relate to a range of events or stressors. After deployment, the symptoms are likely a reaction to the warzone experiences in which life-threatening situations are experienced or witnessed, like being shot at, being exposed to the explosion of an improvised explosive device (IED), or having to help with the removal of human remains. Such experiences can drastically change one's view on the world, like perceiving the world as a dangerous place, and one's evaluative reactions (e.g., Foa and Rothbaum, 1998; Ehlers and Clark, 2000; Engelhard et al., 2009a, 2011). Moreover, common posttraumatic symptoms like having unexpected, distressing thoughts about the event, nightmares, and sleeping problems can be negatively interpreted and may lead to a change in the soldier's view on his/her self, such as “I am a weak person,” or “My reactions since the event mean that I am going crazy” (Foa et al., 1999). The question that arises is whether it is realistic to expect measurement invariance for the situation as described here.

In sum, assessing levels of PTSD symptoms at baseline as well as after the traumatic events is essential to model the development of PTSD symptoms, but may be statistically problematic at the same time because of expected measurement non-invariance.

### This study

In the current study, we tested measurement invariance in two datasets that were part of two larger prospective studies about resilience and vulnerability factors involved in PTSD symptoms (see Lommen et al., 2013 for sample 1, and Engelhard et al., 2007b for sample 2). Using Sample 1, we investigated the source of the measurement non-invariance, including the effect of the presence or absence of prior deployment experiences. Arguably, those with prior deployment experiences are more likely to fill out the questionnaire with regard to deployment related traumatic experiences at both time points. Expecting measurement invariance may therefore be specifically unrealistic for the group without prior deployment experience. Sample 2 was used to test whether the results of sample 1 would be replicated. Finally, solutions for dealing with non-invariant data will be discussed.

## Material and methods

Sample 1 consisted of 249 Dutch soldiers [Task Force Uruzgan (TFU) 11], who completed the Dutch version (Engelhard et al., 2007a) of the Posttraumatic Symptom Scale—Self Report (PSS; Foa et al., 1993) about 2 months before their 4-month deployment to Afghanistan (*N* = 249), and about 2 months after their return home (*n* = 241). The PSS is a self-report questionnaire with 17 items that represent the 17 symptoms of PTSD according to the DSM-IV (American Psychiatric Association, 2000), which includes (a) re-experiencing symptoms, such as intrusions, flashbacks, and nightmares (b) avoidance symptoms (e.g., avoidance of reminders of the traumatic event) and numbing, and (c) hyperarousal symptoms, such as hypervigilance, sleep disturbances, and concentration problems. Before their deployment, participants were asked to rate the questions with respect to their most aversive life-event that troubles them the most in the last month. After deployment, participants were instructed to complete the PSS with respect to their deployment-related event(s) that troubled them the most in the last month. Items were rated on a 0 (*not at all*) to 3 (*almost always*) scale. For convenience, scores were dichotomized into 0 *(symptom absent)* to 1 *(symptom present)* for the analyses.

Sample 2 consisted of 305 Dutch soldiers, derived from a larger study in which 481 soldiers were included [stabilization Force Iraq (SFIR) 3, 4, and 5; Engelhard et al., 2007b]. Since only SFIR 3 and 5 were asked to complete the PSS before their deployment, these two groups were included in this study (*N* = 310). Only soldiers who completed the PSS at least at one of the two time points were included in this study (*n* = 305). Before their deployment to Iraq, 291 soldiers filled out the PSS, and 242 soldiers completed the PSS about 5 months after their return home.

At the post-deployment assessment, both samples completed a Dutch version of the Potentially Traumatizing Events Scale (PTES; Maguen et al., 2004), which assessed the frequency of exposure to war-zone related stressors. For sample 1, the questionnaire was adjusted to the situation in Afghanistan, resulting in 24 stressors (cf. Lommen et al., 2013). For sample 2, the questionnaire was adjusted to the situation in Iraq, resulting in 22 stressors (cf. Engelhard and van den Hout, 2007). Participants indicated whether they had experienced each stressor, and the negative impact (no, mild, moderate, or severe).

Participation was strictly voluntary without financial compensation. Both prospective projects were approved by the Institutional Review Board of Maastricht University.

### Data analysis

Analyses were conducted with Mplus 7.11 (Muthén and Muthén, 2010). First, using Sample 1, two confirmatory factor analyses (CFA) for the PSS at the two time points were assessed. Second, measurement invariance was tested, as suggested by Raykov et al. (2012) by comparing the model fit of four competing, but nested, models: the unconstrained CFA model (factor loadings and thresholds of the latent variable were freely estimated), the CFA model with threshold invariance (constrained thresholds), the CFA model with loading invariance (constrained factor loadings), and the CFA model with scalar invariance (constrained factor loadings and thresholds). The tests for determining measurement invariance were repeated for Sample 2 to investigate whether the results for Sample 1 could be replicated. Third, to investigate whether the measurement invariance test would be different for soldiers with and without prior deployment experiences, the previous step was repeated for these two groups separately. Fourth, to gain insight in the source of potential measurement non-invariance we applied two methods: (1) differences in factor loadings and thresholds were tested using a Wald test; and (2) we employed the method of Raykov et al. (2013). For the first method we used the loading invariance model and tested each pair of thresholds using the MODEL TEST option in Mplus. This procedure resulted in 17 Wald tests. For the second method, of Raykov et al., we first tested the chi square difference (using the DIFFTEST option of Mplus) between the scalar model and 17 models (17 items) where one pair of thresholds was left unconstrained at a time (Method 2A). This resulted in 17 chi square difference tests. If all tests in comparison to the scalar model are non-significant, then measurement invariance holds. If some tests are significant whereas others are not, we can conclude that partial invariance holds and we know which items are causing the non-invariance. Since the CFA models indicated that the loading invariance model showed the best fit (with thresholds freely estimated), we also computed the chi difference tests between the loading invariance model and 17 models where one set of thresholds was constrained (Method 2B). This latter procedure is a replication of the first method, with the MODEL TEST option, but this time with chi square values instead of Wald tests. The two methods (i.e., 2A and 2B) can be considered as the forward and backward methods of sequential regression analyses and will probably result in slightly different solutions just like with sequential analyses.

For the Raykov method we applied the Benjamini-Hochberg multiple testing procedure as described in Raykov et al. (2013). That is, we calculated a corrected alpha value, indicated by *l* in the tables. The *p*-values of the chi square difference tests should then be smaller than *l* instead of the default alpha of.05. After computing the chi square differences, the resulting *p*-values are ordered from small to large and for each row a different *l* value is computed. For more details, how to compute *l* and syntax-examples we refer to Raykov et al. (2013). In the appendix of our paper we provide our Mplus syntax for the final model of method 1 (all other syntax files can be found at the website of the second author: http://www.rensvandeschoot.com) and in the footnote of **Table 3** we provide the code for obtaining *l*.

The root mean square error of approximation (RMSEA, Steiger, 1990), comparative fix index (CFI; Bentler, 1990), and Tucker-Lewis index (TLI; Tucker and Lewis, 1973) were used to evaluate model fit. RMSEA values of <0.08, CFI, and TLI values of >0.90 were considered to reflect adequate model fit (see Kline, 2010 for an overview of fit statistics). To compare models, we used Chi square difference test, Akaike Information Criterion (AIC; Akaike, 1981) and Bayesian Information Criterion (BIC; Schwarz, 1978) values.

## Results

### Experienced events on deployment

The most commonly experienced deployment-related events in all samples (TFU 11 of sample 1, SFIR 3 and SFIR 5 of sample 2) were “Going on patrols or performing other dangerous duties” (90–94%), “Fear of being ambushed or attacked” (65–95%), and “Fear of having unit fired on” (61–95%). Amongst those events that participants rated as having a moderate to severe negative impact were “Being informed of a Dutch soldier who got killed” (21–51%), “Witnessing an explosion” (9–25%), “Seeing dead or injured Dutch soldiers” (0–24%), and “Having to aid in the removal of human remains” (0–13%).

### Sample 1

CFA models including the latent variable PSS loading on 17 indicators showed acceptable model fit at both time points [before deployment: χ^2^_(119)_ = 175.027, *p* < 0.001, RMSEA (90% CI) = 0.044 (0.029–0.058), CFI = 0.961, TLI = 0.955; after deployment: χ^2^_(119)_ = 175.237, RMSEA (90% CI) = 0.044 (0.029–0.058), CFI = 0.921, TLI = 0.909]. Table 1 presents an overview of the fit indices used to evaluate the CFA-models including PSS at both time points. The CFA including PSS at both time points with freely estimated factor loadings and the CFA with loading invariance showed acceptable model fit. The model fit of the unconstrained CFA was better according to the chi square difference test, CFI, TLI, and RMSEA, but the CFA with loading invariance (see Appendix 1 for Mplus syntax of model statement) was better according to the AIC and BIC. The CFA that imposed threshold invariance and the one imposing scalar invariance both showed unacceptable model fit. The results of all fit indices indicate that the measurement non-invariance has mainly to do with the instability of the thresholds over time.

**Table 1:** **Model fit information for CFA including PSS before and after deployment in sample 1 and 2**.

	**χ^2^(df)**	**CFI**	**TLI**	**RMSEA (90% CI)**	**AIC**	**BIC**
**SAMPLE 1**						
Unconstrained	640.821 (526)	0.924	0.919	0.030 (0.020–0.037)	5974.361	6217.065
Threshold invariance	751.535 (543)	0.862	0.857	0.039 (0.032–0.046)	6034.422	6217.330
Loading invariance	674.540 (543)	0.913	0.910	0.031 (0.023–0.039)	5965.915	6148.823
Scalar invariance	772.401 (560)	0.859	0.859	0.039 (0.032–0.046)	6218.945	6342.056
**SAMPLE 2**						
Unconstrained	630.235 (526)	0.961	0.959	0.025 (0.017–0.033)	6639.398	6896.100
Threshold invariance	763.777 (543)	0.918	0.915	0.037 (0.030–0.042)	6715.873	6909.330
Loading invariance	618.640 (543)	0.972	0.971	0.021 (0.011–0.029)	6621.558	6815.014
Scalar invariance	726.491 (560)	0.938	0.938	0.031 (0.024–0.037)	6830.930	6961.140

*AIC and BIC through MLR, rest: WLSMV*.

### Sample 2

Similar to sample 1, the CFA models including the latent variable PSS in sample 2 showed acceptable model fit at both time points [before deployment: χ^2^_(119)_ = 160.476, *p* = 0.007, RMSEA (90% CI) = 0.035 (0.019–0.048), CFI = 0.941, TLI = 0.933; after deployment: χ^2^_(119)_ = 219.654, RMSEA (90% CI) = 0.059 (0.047–0.071), CFI = 0.963, TLI = 0.957]. Although in this sample all CFA models with varying constrains showed acceptable model fit, AIC and BIC were lowest for the loading invariance model (see Table 1). Again, the measurement non-invariance seems to arise from instability of the thresholds.

### Prior deployment experience

It could be argued that measurement non-invariance would be driven by those participants who have not been deployed before, because they may refer to different types of stressors before and after this particular deployment when rating the items. For those participants who have been deployed before, the meaning of the construct might have already changed with the experience of the prior deployment. Therefore we tested measurement invariance in the group with (56.63 and 41.64% in Sample 1 and 2, respectively) and without prior deployment experience separately. Nevertheless, based on AIC/BIC comparison, the results showed a similar pattern for both groups, suggesting that threshold instability underlies measurement non-invariance in our samples, regardless of the presence or absence of prior deployment experience. The results can be found in the online available supplementary materials.

### Threshold instability

To gain insight in the instability of the thresholds for both samples, we explored the difference in thresholds for each item between the two time points. For descriptive purposes, the threshold before deployment was subtracted from the threshold after deployment difference to define threshold difference for each item. The threshold represents the mean score on the latent variable that is related to the “turning point” where an item is rated as present instead of not present. Thus, a positive difference score means that compared to the PSS mean score before deployment, a higher PSS mean score was needed to rate an item as present after deployment. Threshold values and difference scores are presented in Table 2.

**Table 2:** **Threshold and threshold difference (threshold after deployment minus threshold before deployment) per item of the Posttraumatic Symptom Scale—Self Report (PSS)**.

**THRESHOLD**						
**Item**	**Sample 1**			**Sample 2**		
	**Pre**	**Post**	**Diff**	**Pre**	**Post**	**Diff**
1. Recurrent and intrusive distressing recollections of the event	0.221	1.411	1.190^*^	0.895	0.908	0.049
2. Recurrent distressing dreams of the event	1.440	1.130	−0.310^*^	1.462	0.990	−0.472^*^
3. Acting or feeling as if the event were recurring	1.054	1.306	0.252	1.005	0.940	−0.065
4. Intense psychological distress at exposure to cues of event	1.036	1.569	0.533^*^	1.820	1.060	−0.760^*^
5. Physiological reactivity on exposure to cues of event	1.258	1.643	0.385^*^	1.264	1.135	−0.129
6. Avoidance of thoughts, feelings, or conversations associated with event	0.623	1.836	1.213^*^	1.435	0.762	−0.673^*^
7. Avoidance of activities, places, or people associated with event	1.036	1.647	0.611^*^	1.345	1.415	0.070
8. Inability to recall an important aspect of event	0.919	1.356	0.437^*^	1.191	1.197	0.006
9. Diminished interest or participation in significant activities	0.801	1.021	0.220	1.209	0.668	−0.541^*^
10. Feeling of detachment or estrangement from others	0.987	1.216	0.229	1.191	0.776	−0.415^*^
11. Restricted range of affect	1.113	0.890	−0.223^*^	0.869	0.630	−0.239^*^
12. Sense of a foreshortened future	1.019	1.359	0.340^*^	1.017	1.385	0.368^*^
13. Difficulty falling or staying asleep	0.921	0.830	−0.091	0.820	0.665	−0.155
14. Irritability or outbursts of anger	0.258	0.221	−0.037	0.856	0.273	−0.583^*^
15. Difficulty concentrating	0.552	0.745	0.193	0.650	0.655	0.005
16. Hypervigilance	0.830	0.330	−0.500^*^	1.245	−0.166	−0.411^*^
17. Exaggerated startle response	1.608	0.704	−0.904^*^	0.694	0.484	−0.210

* *p < 0.05*.

The first method we used to test for threshold differences is to compute a Wald test whether, for each item, the threshold after deployment significantly increased or decreased compared to the threshold before deployment. As can be seen in Table 2, where significant differences are indicated with an asterisk, the majority of the threshold values changed significantly (11 and 9 out of the 17 thresholds for sample 1 and 2, respectively). A decrease in threshold means that the possibility of answering “yes” after deployment was higher than the possibility of a “yes” before deployment, whereas the possibility of answering “yes” was lower after deployment compared to before deployment for those thresholds that increased. According to this method, four items changed significantly in the same direction in both samples: thresholds for “Recurrent distressing dreams of the event,” “Restricted range of affect,” and “Hypervigilance” decreased, while “Sense of foreshortened future” increased. Only the threshold of three items (i.e., “Acting or feeling as if the event were recurring,” “Difficulty falling or staying asleep,” and “Difficulty concentrating”) did not change significantly in either sample.

The second method was based on chi square differences between either the scalar (method 2A; see Table 3) or the loading invariance model (method 2B; see Table 4) and 17 models where one combination of thresholds is released or fixed, respectively. Method 2A showed more items with stable thresholds over time, but there was almost no overlap on item level between the two samples. The results of method 2B were similar to the results of method 1, with the only difference that some item thresholds that significantly changed over time according to method 1, did not significantly change according to the *l* value, but only when a *p* value of.05 was used.

**Table 3:** **Chi square difference values, p-, and l-values for the scalar model where the model number refers to the item number of which the thresholds between the two time points is estimated unconstrained (all factor loadings and other thresholds are constrained)**.

**Sample 1**			**Sample 2**			**I**
**Model**	**χ^2^**	***p***	**Model**	**χ^2^**	***p***	
M1	77.719	<0.0001^*^	M16	106.308	<0.0001^*^	0.00085
M2	17.674	<0.0001^*^	M12	29.885	<0.0001^*^	0.00171
M17	54.284	<0.0001^*^	M15	18.237	<0.0001^*^	0.00256
M6	48.995	<0.0001^*^	M6	9.874	0.001^*^	0.00342
M16	45.051	<0.0001^*^	M14	9.741	0.001^*^	0.00427
M11	15.203	0.001^*^	M4	9.139	0.002^*^	0.00513
M7	9.590	0.002^*^	M7	7.512	0.006^**^	0.00598
M4	7.017	0.008^**^	M8	6.412	0.011^**^	0.00684
M14	6.755	0.009^**^	M9	5.176	0.022^**^	0.00769
M13	6.493	0.011^**^	M5	4.235	0.039^**^	0.00855
M8	5.450	0.020^**^	M3	3.935	0.047^**^	0.00940
M5	3.146	0.076^***^	M13	3.363	0.066^***^	0.01026
M12	2.296	0.130^***^	M2	2.789	0.094^***^	0.01111
M3	1.477	0.224^***^	M17	1.156	0.282^***^	0.01197
M10	1.128	0.288^***^	M10	0.580	0.446^***^	0.01282
M9	1.088	0.297^***^	M11	0.485	0.486^***^	0.01368
M15	0.005	0.942^***^	M1	0.005	0.941^***^	0.01453

* *significant when p ≤ l*.

** *significant when p ≤ 0.05*.

*** *never significant*.

l = {0.05/[17^*^(1+1/2+1/3+1/4+1/5+1/6+1/7+1/8+1/9+1/10+1/11+1/12+1/13+1/14+1/15+1/16+1/17)]^*^c where c = 1,…,17 to obtain a new alpha value for each new test.

**Table 4:** **Chi square difference values, p-, and l-values for the loading invariance model where the model number refers to the item number of which the thresholds between the two time points is constrained (all factor loadings are constrained and other thresholds are unconstrained)**.

**Sample 1**			**Sample 2**			**I**
**Model**	**χ^2^**	***p***	**Model**	**χ^2^**	***p***	
M1	92.568	<0.0001^*^	M16	130.2250	<0.0001^*^	0.00085
M6	56.579	<0.0001^*^	M14	27.0260	<0.0001^*^	0.00171
M16	22.125	<0.0001^*^	M6	23.6180	<0.0001^*^	0.00256
M17	35.555	<0.0001^*^	M9	21.8750	<0.0001^*^	0.00342
M7	13.277	<0.0001^*^	M4	21.0990	<0.0001^*^	0.00427
M4	11.135	0.001^*^	M10	13.6190	<0.0001^*^	0.00513
M8	9.798	0.002^*^	M2	13.4300	0.001^*^	0.00598
M5	5.807	0.016^*^	M12	8.4590	0.003^*^	0.00684
M12	5.232	0.022^**^	M11	5.9620	0.014^**^	0.00769
M2	4.890	0.027^**^	M17	4.3380	0.037^**^	0.00855
M11	3.969	0.046^**^	M13	1.8990	0.168^***^	0.00940
M15	3.960	0.046^**^	M3	1.2580	0.262^***^	0.01026
M9	3.890	0.048^**^	M5	1.0110	0.314^***^	0.01111
M10	3.497	0.061^***^	M15	1.0020	0.316^***^	0.01197
M3	2.777	0.095^***^	M7	0.2040	0.651^***^	0.01282
M14	1.132	0.287^***^	M1	0.1580	0.690^***^	0.01368
M13	0.607	0.436^***^	M8	0.0020	0.963^***^	0.01453

* *significant when p ≤ l*.

** *significant when p ≤ 0.05*.

*** *never significant*.

l = 0.05/[17^*^(1+1/2+1/3+1/4+1/5+1/6+1/7+1/8+1/9+1/10+1/11+1/12+1/13+1/14+1/15+1/16+1/17)]^*^c where c = 1,…,17 to obtain a new alpha value for each new test.

In sum, the three methods resulted in different items being problematic and not all items were similarly problematic across the two samples. Looking at the subscales of the PSS (subscales according to the DSM-IV and psychometric studies), each subscale included one or more unstable items. So the main conclusion is that the instrument assessing posttraumatic stress symptoms has way too many non-invariant items to justify latent mean comparison over time.

## Discussion

To compare latent mean scores over time, the latent variable should be measurement invariant. However, it might not always be realistic to expect measurement invariance. In the current study we tested whether the underlying construct of a posttraumatic stress questionnaire changed over time by the experience of a traumatic event. This change seems likely, since such a major life experience challenges someone's beliefs about others, the world, and themselves (e.g., Foa and Rothbaum, 1998; Ehlers and Clark, 2000). At the same time, however, assessment of posttraumatic stress before and after a traumatic event is important to study the development op posttraumatic stress disorder after a specific event; that is, already existing symptoms should be taken into account. In the present study, measurement invariance of the posttraumatic symptom scale (PSS; Foa et al., 1993) was tested in two samples of Dutch soldiers who completed the PSS before and after deployment.

According to our first statistical method, results from our test for measurement invariance in Sample 1 showed instability of the thresholds of almost all indicators (the items). Analyses in Sample 2 replicated these findings, but other indicators appeared to be causing the non-invariance. Results were also similar when only those soldiers with or without prior deployment experience were included. Taking both samples into account, only 3 item thresholds showed no significant changes over time. The instability of thresholds was replicated with two other statistical methods, although not all thresholds were similarly problematic across the different methods and the two samples. Since the lack of measurement invariance is due to threshold instability of the majority of the items, it seems reasonable to conclude that the underlying construct of PSS is unstable over time if war-zone related traumatic events occur in between measurements. This finding might also explain the lack of measurement (scalar) invariance found in a study that compared soldiers who had or had not been recently deployed (Mansfield et al., 2010).

From a statistical viewpoint, based on the findings of this study it could be argued that *any* PTSD-related questionnaire is expected to fail the test for measurement invariance. As a result, measurement invariance should never be taken for granted, but should be tested. Moreover, if non-invariance is found, an increase or a decrease of PSS cannot be interpreted in a straightforward way in a prospective longitudinal study in which the PSS is assessed before and after trauma e.g., using, longitudinal models like repeated measure analyses or latent growth (mixture) models. One solution is to treat the pre-trauma assessment as a different construct. Giving the constructs before and after the traumatic event different names can emphasize this: the pre-deployment score could be named “baseline symptoms” (Lommen et al., 2014) and the post-deployment score could be named “PTSD symptoms.”

A few points should be taken into consideration with regard to this study. First, although we cross-validated our results in two samples and with different statistical methods, the findings should be replicated in samples from different countries to exclude country specific effects. Also, the results should be replicated in samples with different DSM-classified traumatic events to find out whether the results are specific for military forces or that the results can be generalized to all traumatic events. Moreover, other, more efficient, methods of detecting non-invariant items could be used (de Roover et al., 2014), but at least our conservative method of pairwise testing provides a first step. Future studies may focus on identifying more stable items to construct a questionnaire to use in prospective studies that include measurements before and after trauma exposure. Second, in this study, PTSD was used as a latent construct. The idea that PTSD symptoms are indicators of an underlying latent variable is widespread. According to this view, the PTSD construct denotes a latent variable that functions as the root cause of PTSD symptoms. This presumption has directed psychopathology research for decades, but rests on problematic psychometric premises (Borsboom and Cramer, 2013; McNally et al., in press). Recently, alternative, network approaches have been proposed that conceptualize mental disorders as systems of causally connected symptoms (Borsboom and Cramer, 2013; McNally et al., in press). Future studies might investigate change in PTSD symptoms from a network approach perspective.

### Recommendations

Our advice for PTSD researchers who use PTSD as a latent construct in pre-trauma and post-trauma designs is to always test for measurement invariance for measures. Since measurement non-invariance is highly likely to be found if a traumatic event occurred in between two assessments, it is important to investigate the source of the construct instability, and treat the pre and post scores as different construct for each time point in the analysis.

### Conflict of interest statement

The authors declare that the research was conducted in the absence of any commercial or financial relationships that could be construed as a potential conflict of interest.

## Footnotes

^1^Exposure to actual or threatened death, serious injury or sexual violation. The exposure must result from one or more of the following scenarios, in which the individual:

- directly experiences the traumatic event;
- witnesses the traumatic event in person;
- learns that the traumatic event occurred to a close family member or close friend (with the actual or threatened death being either violent or accidental); or
- experiences first-hand repeated or extreme exposure to aversive details of the traumatic event (not through media, pictures, television or movies unless work-related).
