# Supplementary material for: The experience of traumatic events disrupts the measurement invariance of a posttraumatic stress scale
Source: Front Psychol. 2014 Nov 18;5:1304. doi: 10.3389/fpsyg.2014.01304 (PMC4235410; doi:10.3389/fpsyg.2014.01304)
Supplement: Supplementary file 1 [file DataSheet1.DOCX]

APPENDIX 1.

Mplus model statement of loading invariance model

MODEL:

PSSpre BY pre1-pre17* (a1-a17);

PSSpost BY post1-post17* (a1-a17);

PSSpost ON PSSpre;

[PSSpre@0];

[PSSpost@0];

PSSpre@1;

PSSpost@1;
